# Improvement of Intestinal Pseudo-Obstruction by Total Parenteral Nutrition in a Young Woman With Mitochondrial Myopathy, Encephalopathy, Lactic Acidosis, and Stroke-Like Episodes: A Case Report

**DOI:** 10.7759/cureus.50075

**Published:** 2023-12-06

**Authors:** Naoto Sakamoto, Shuhei Hamada, Hiroki Takahashi, Rumi Satou, Masatsune Suzuki, Tetsuhiro Maeno

**Affiliations:** 1 Department of Primary Care and Medical Education, Institute of Medicine, University of Tsukuba, Tsukuba, JPN; 2 Department of General Medicine, Taito Municipal Hospital, Tokyo, JPN; 3 Department of Internal Medicine, Kamisu Saiseikai Hospital, Kamisu, JPN

**Keywords:** incretin-related drugs, conservative therapy, total parenteral nutrition, intestinal pseudo-obstruction, melas

## Abstract

Patients with mitochondrial myopathy, encephalopathy, lactic acidosis, and stroke-like episodes (MELAS), a mitochondrial disease, develop various types of organ failure, including intestinal pseudo-obstruction (IPO). We treated a patient with IPO that improved with total parenteral nutrition. A 20-year-old woman with a two-year history of diabetes mellitus was taking sitagliptin but her hemoglobin A1c (HbA1c) levels began increasing. After receiving metformin, she suffered a stroke-like attack and was diagnosed with MELAS. After persistent anorexia, she presented with symptoms of IPO, such as vomiting and gastrointestinal dilatation.

After about 10 days of total parenteral nutrition, intestinal peristalsis improved and bowel movements resumed. She was able to resume her normal diet, and glycemic control with insulin glargine has allowed her to return to her daily life without gastrointestinal symptoms for over six months. Total parenteral nutrition may be effective for MELAS with IPO, and good glycemic control can prevent the need for incretin-related drugs, thus reducing the likelihood of recurrent IPO.

## Introduction

Mitochondrial myopathy, encephalopathy, lactic acidosis, and stroke-like episodes (MELAS) is a mitochondrial disease that causes dysfunction of various organs [1]. One of these is intestinal pseudo-obstruction (IPO), a clinical and anatomic syndrome characterized by impaired peristalsis and dilation of the intestinal tract not caused by organic stenosis or inflammation [2]. 

The following four diagnostic criteria for IPO have been proposed: 1) one or more symptoms of ileus, with onset at least six months prior to diagnosis; 2) abdominal pain and/or bloating for the past 12 weeks; 3) dilatation and/or niveau of the intestine on abdominal X-ray, echo, and/or computed tomography imaging; and 4) no evidence of structural disease that could explain dilatation and/or niveau of the intestine, including findings on upper endoscopy, lower endoscopy, computed tomography, barium enema, and small-bowel follow-through [3]. The A3243G point mutation, found in 80% of patients with MELAS, causes impaired mitochondrial energy production [1]. 

It has been suggested that the pathophysiological mechanism of IPO is likely related to mitochondrial dysfunction and imbalances in energy metabolism [4]. Thus, nutritional management of IPO in patients with MELAS might contribute to treatment when mitochondrial function remains and energy supply is inadequate.

Conservative pharmacological treatment of IPO in patients with MELAS using laxative agents, antidiarrheal drugs, and antiflatulence agents was common in previous reports [4,5], but their efficacy is unclear because they do not contribute to mitochondrial function, which is thought to cause IPO. The only report that mentions the effect of nutritional therapy with total parenteral nutrition (TPN) involved a single patient with MELAS who had an ileostomy constructed [6]. Surgical treatment of IPO in patients with MELAS has been associated with a poor prognosis [7], and therefore treatment that avoids surgery is desirable. The optimal management of patients with MELAS to prevent IPO recurrence is unknown.

Here we present a young woman with MELAS who had no previous surgery and who developed IPO shortly after diagnosis, which improved quickly with early initiation of TPN.

## Case presentation

We report a patient with IPO due to MELAS who recovered successfully with TPN and who developed no IPO recurrence for a long period of time. 

A 20-year-old woman with MELAS was transferred to our hospital for continued rehabilitation for persistent anorexia, aphasia, and gait disturbance. She had an intellectual disability and short stature, and was diagnosed with type 2 diabetes two years ago. She had been taking sitagliptin 50 mg/day since the diagnosis of diabetes, and her hemoglobin A1c (HbA1c) had improved to 5.9%. More recently, however, her HbA1c had gradually been increasing, and therefore her sitagliptin was increased to 100 mg/day three months ago. Nonetheless, her HbA1c worsened to 8.1%, even though her diet remained the same. We therefore also prescribed metformin at a dosage of 500 mg/day two months ago. 

**Figure 1:**
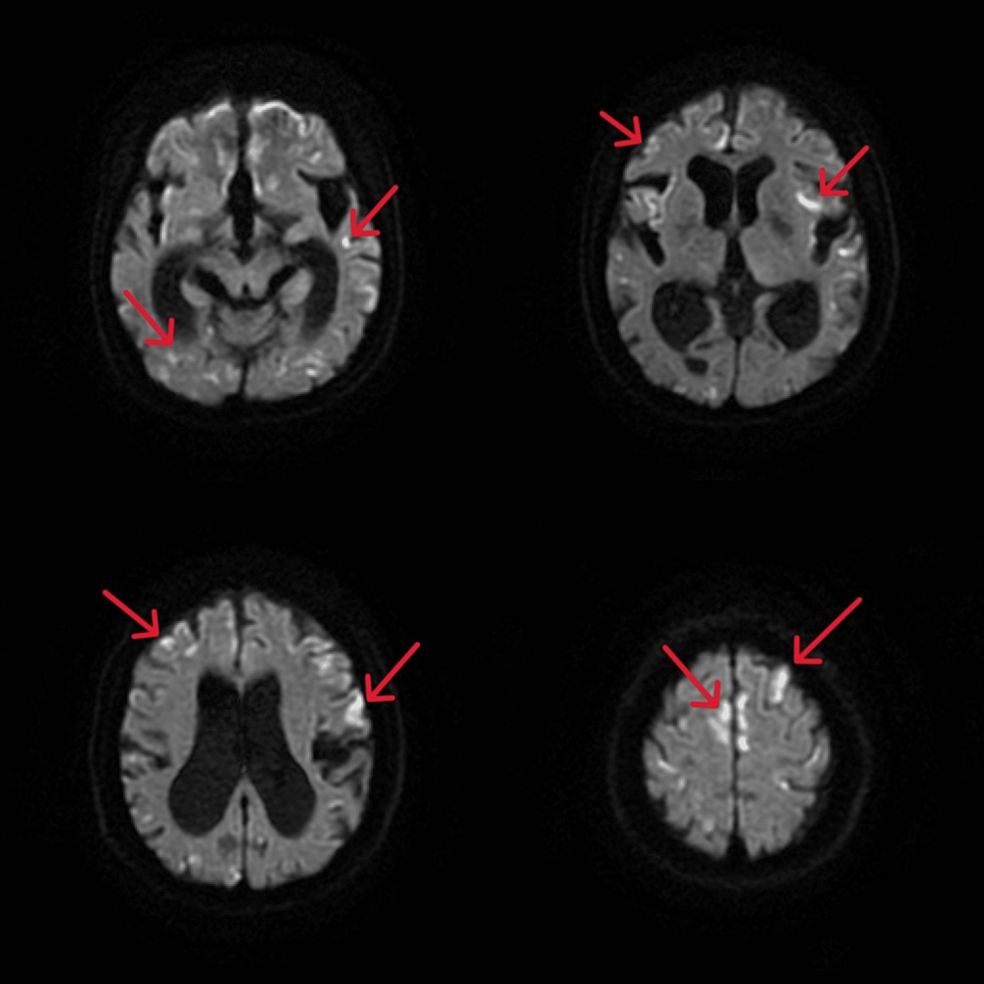
Head MRI showed new scattered cerebral infarcts. Red arrows show cortical and subcortical infarcts. Frontal lobe infarcts are also present.

Three days after starting metformin, she developed vomiting, difficulty with oral intake, and tonic seizure, and she was hospitalized because of anorexia and vomiting. An abdominal X-ray showed no niveau formation or intestinal dilatation. While hospitalized, she developed impaired consciousness, tonic seizure, and vomiting, which together resulted in lactic acidosis, aphasia, and gait disturbance. Head MRI showed new scattered cerebral infarcts (Figure 1). 

We ascertained from her family history that several maternal relatives had died suddenly in their 30s, which was one reason to suspect maternally inherited MELAS. She was transferred to another hospital one month ago and underwent genetic testing. Eighty percent of leukocyte mitochondrial DNA was found to contain the A3243G point mutation.

In 1992, clinical guidelines for diagnosing MELAS syndrome were published, and consisted of the following three invariant criteria: 1) stroke-like episodes before age 40 years, 2) encephalopathy characterized by seizures and/or dementia, and 3) mitochondrial myopathy distinguished by lactic acidosis and/or ragged-red fibers. The diagnosis is also considered confirmed if at least two of the following criteria are met: 1) normal early psychomotor development, 2) recurrent headaches, and 3) recurrent vomiting episodes [1,8]. The MELAS study group committee in Japan published a statement indicating that the diagnosis is considered definitive in the presence of at least two category A criteria (headaches with vomiting, seizures, hemiplegia, cortical blindness, and acute focal lesions in neuroimaging) and two category B criteria (high plasma or cerebrospinal fluid (CSF) lactate, mitochondrial abnormalities in muscle biopsy, and a MELAS-related gene mutation)[9].

Our patient was diagnosed with MELAS based on the following Japanese diagnostic criteria [9]: the presence of acute focal lesions on neuroimaging, a history of lactic acidosis and tonic seizure, and the A3243G point mutation in mitochondrial DNA. Physical examination and blood tests at the time of transfer revealed the following: height, 142.2 cm; body mass index (BMI), 12.6 kg/m2; blood urea nitrogen (BUN), 40.4 mg/dl; creatinine, 0.72 mg/dl; and serum lactate, 6.4 mg/dl (normal range 4.0-16.0 mg/dl). She was taking taurine, vitamin B12, folic acid, ubidecarenone, L-arginine, and ascorbic acid via nasogastric tube at the previous hospital because her impaired consciousness prevented her from taking these prognostically beneficial drugs [10] orally. The administration of insulin degludec 2 units per day stabilized her preprandial blood glucose level at about 100-120 mg/dl. She had a persistent and slight decline in appetite, as well as regular bowel movements without nausea, vomiting, or abdominal pain. Despite efforts to promote her dietary intake, she became irritated and selectively consumed only her preferred foods, resulting in limited calorie intake. She frequently declined to undergo blood tests, with aggressive verbal expressions. We attributed her abnormal behavior to the residual effects of a stroke-like episode induced by MELAS. Consequently, we continued to observe her recovery throughout the rehabilitation process. 

**Figure 2:**
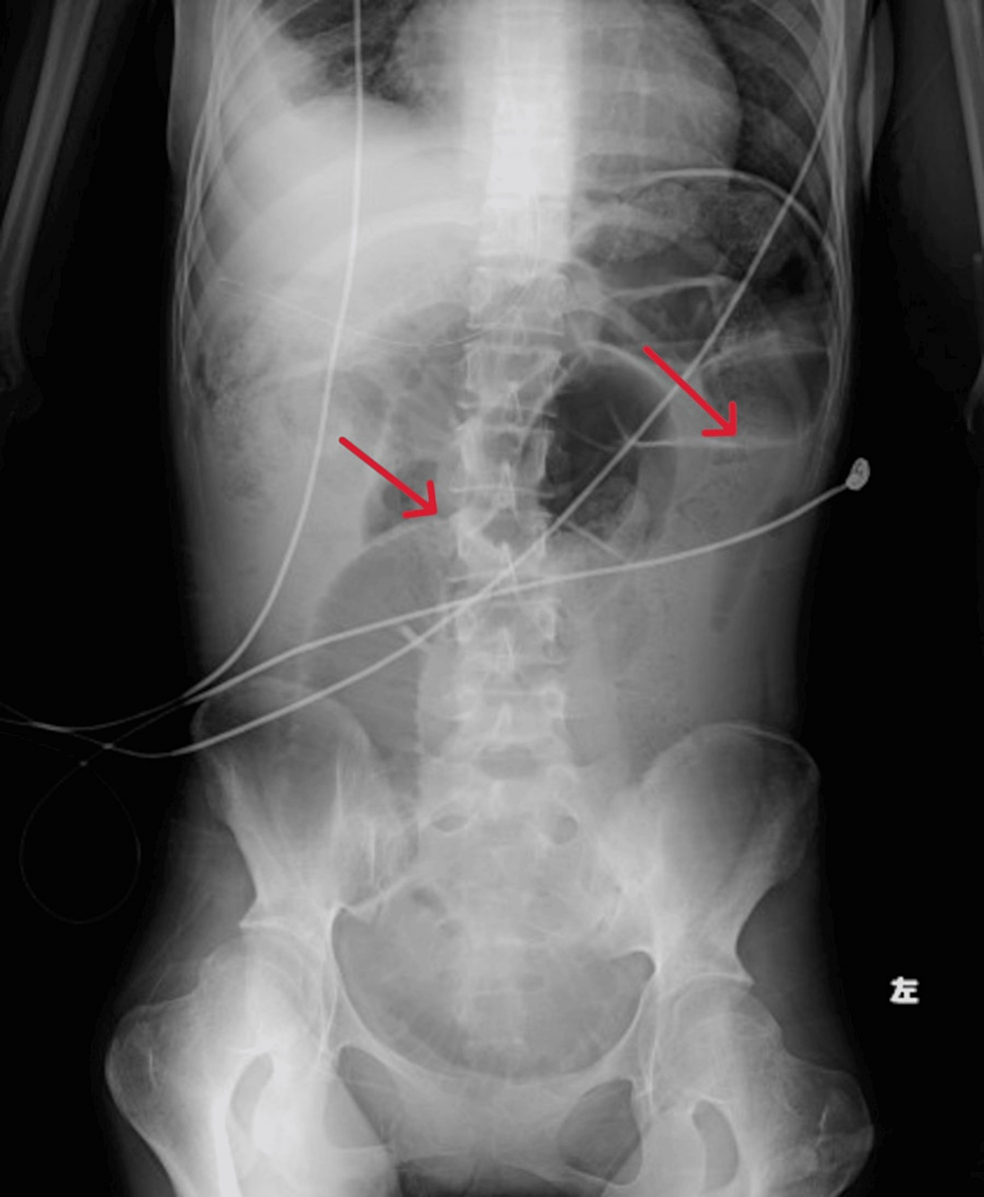
On day 31 after admission she had frequent vomiting and an abdominal X-ray showed marked dilatation of the intestinal tract.

**Figure 3:**
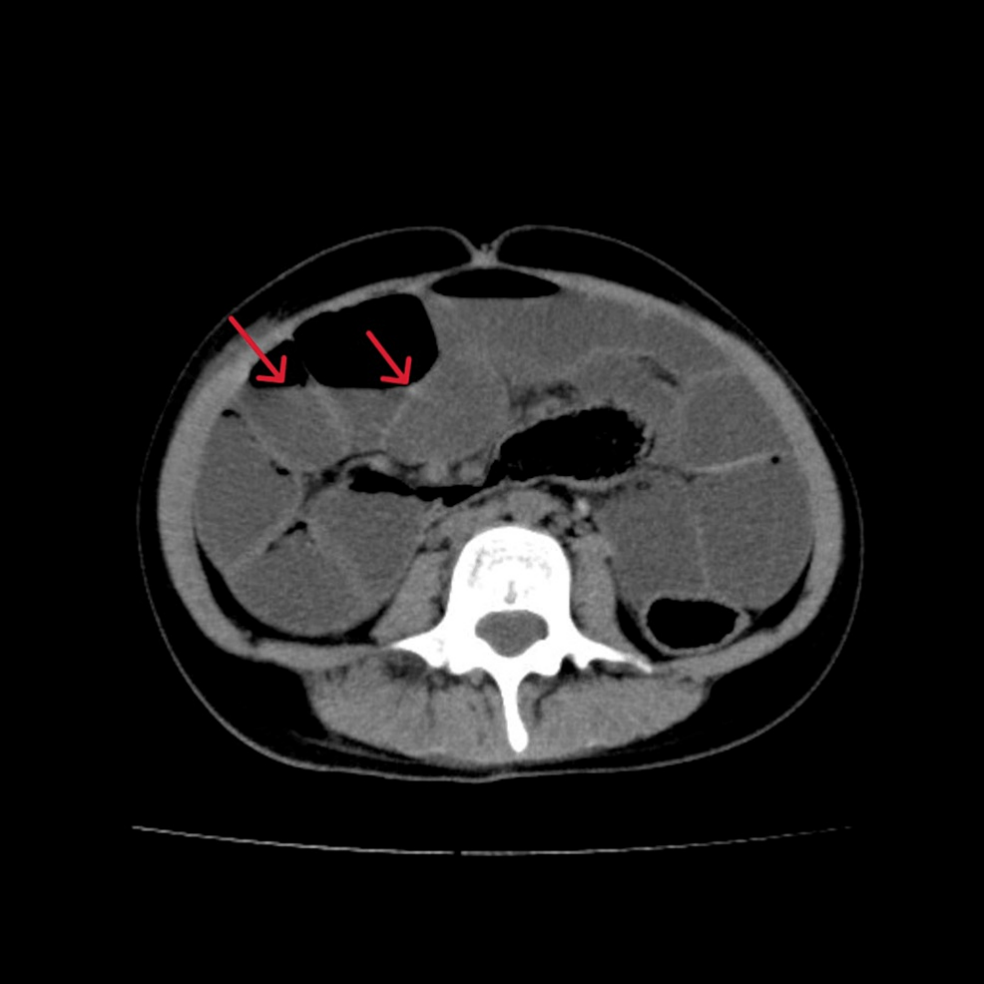
Plain CT of the abdomen shows no ascites, free air, intestinal axis torsion, or intestinal stenosis. Red arrows show niveau formation.

**Figure 4:**
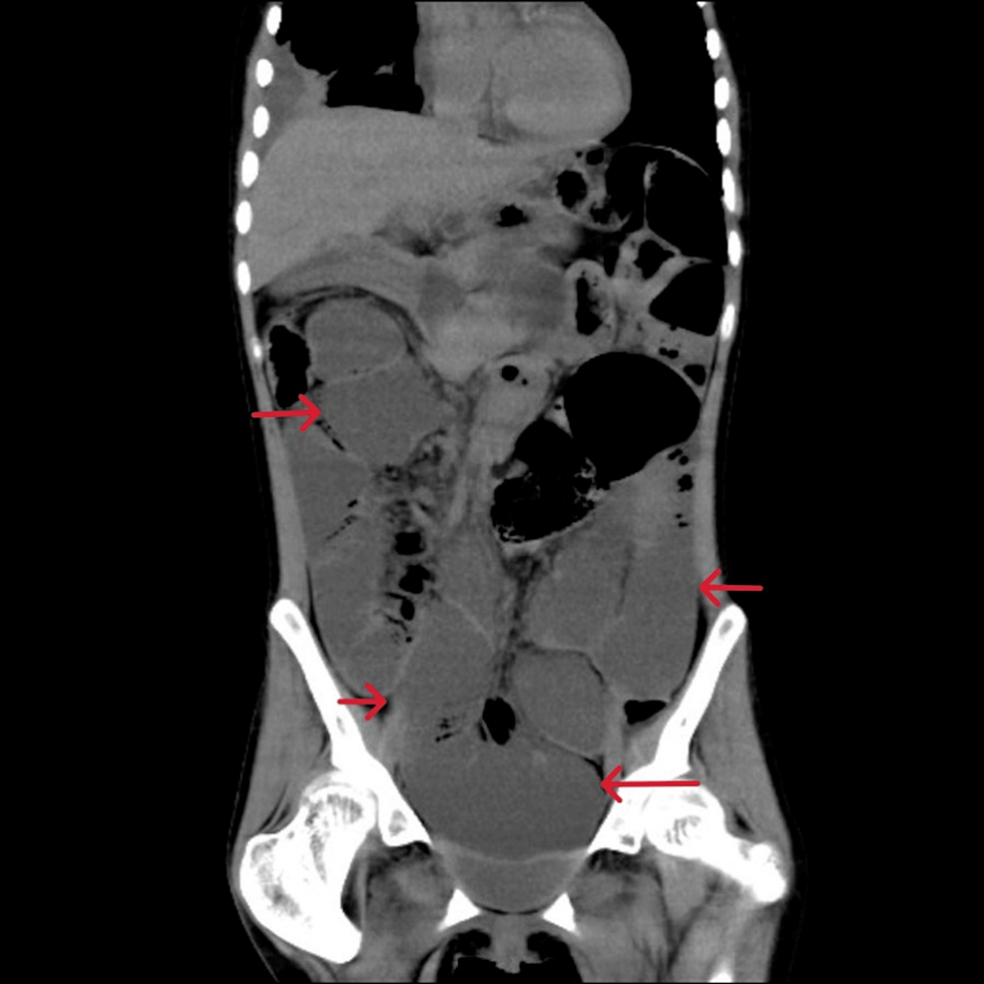
Plain CT of the abdomen (Coronal). Red arrows show dilated, fluid-filled small bowel loops.

On days 13 to 20 after admission, her average daily caloric intake was less than 300 kcal/day. We obtained X-rays and found gas but no niveau formation. On day 21 after admission she complained of malaise. Blood gas analysis revealed lactic acidosis with a serum lactate level of 56.1 mg/dl, and a BUN level of 85.8 mg/dl indicated severe dehydration, so we began a saline infusion. At that time, she had no signs or laboratory findings of conditions that could cause paralytic ileus, such as pancreatitis, cholecystitis, and appenditis. Her caloric intake remained consistently 200-300 kcal/day throughout her admission. On day 29 after admission, she suddenly began eating hardly any food at all. On day 31 after admission she developed frequent vomiting, and an abdominal X-ray (Figure 2) showed marked dilatation of the intestinal tract. We diagnosed her with paralytic ileus, as CT revealed no obstruction (Figures 3, 4).

**Figure 5:**
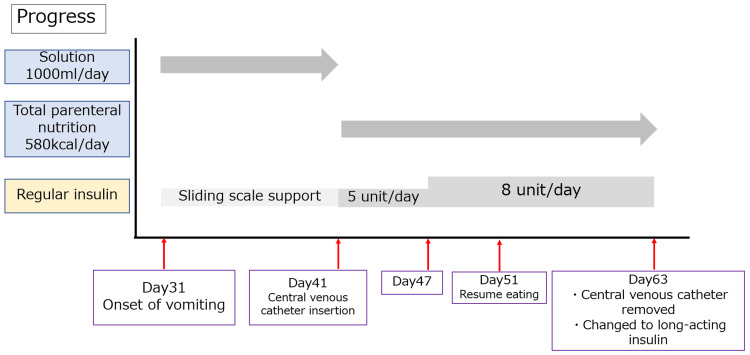
The progress of the case

The diagnostic criteria for chronic intestinal pseudo-obstruction (CIPO) [3] were met, except for the persistence of symptoms, so we diagnosed her with IPO. We reinserted a nasogastric tube and decided on a conservative treatment plan. The progress of the case is shown in Figure 5. 

We did not attribute the paralytic ileus to drugs because we discontinued all oral medications. We initiated peripheral venous supplementation, and on day 33 after admission the patient’s lactate level improved to 21.9 mg/dl, and we therefore did not consider lactic acidosis to be the cause of her paralytic ileus. It did not resolve, and on the 41st day after admission, we started TPN using insulin mixed with a high-calorie infusion formula (580 kcal/day). 

**Figure 6:**
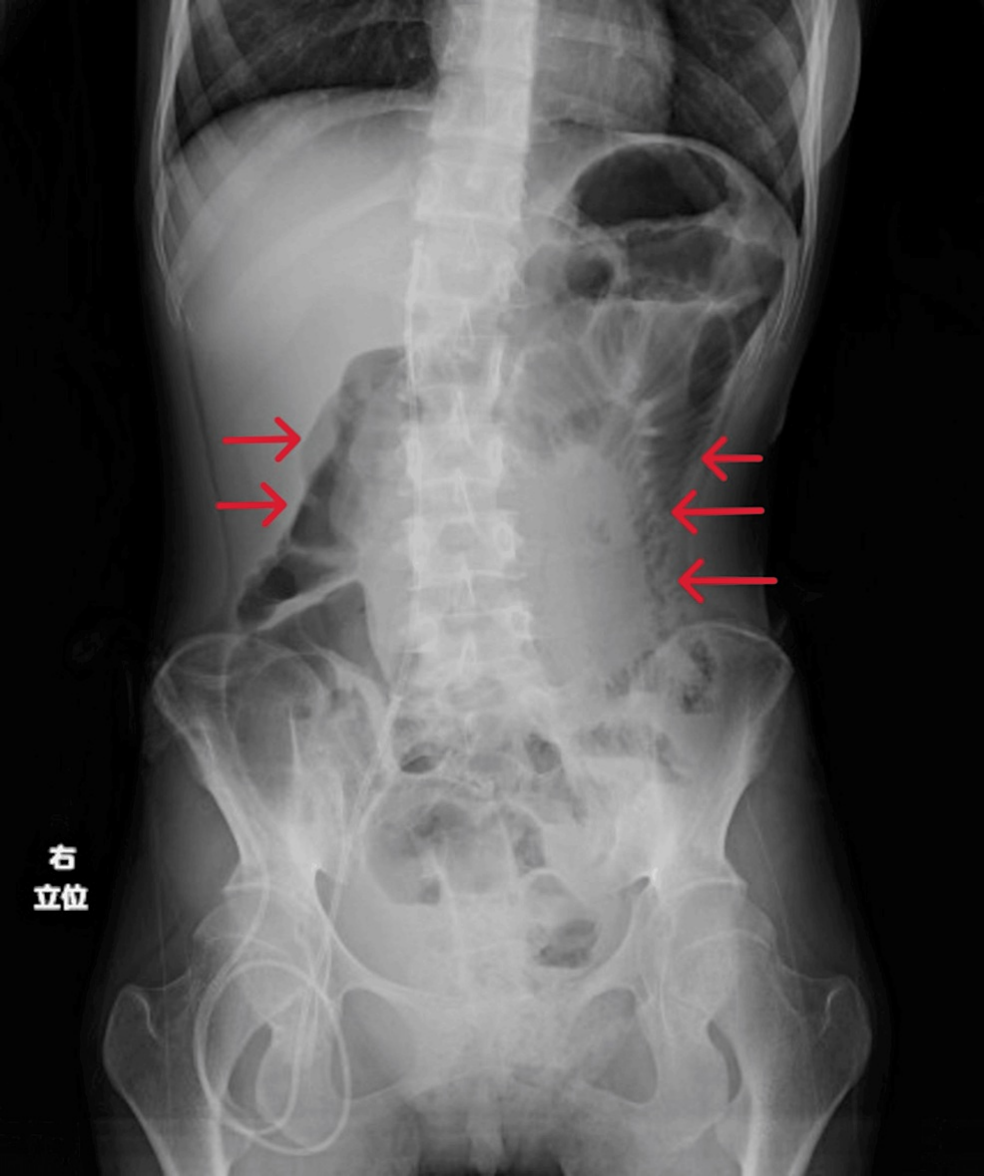
We determined that the paralytic ileus had resolved, and therefore reinitiated oral intake.

On the 10th day of TPN (51st day after admission), she resumed voiding and defecating. We determined that the paralytic ileus had resolved (abdominal X-ray shown in Figure 6), and therefore reinitiated oral intake. 

Through rehabilitation, the severity of her aphasia and gait disorder recovered to that present before admission. Self-injectable insulin rather than oral DPP-4 was initiated because an incretin-based agent could induce bowel obstruction [11]. Insulin administration improved her appetite and achieved stable glycemic control. She had no recurrence paralytic ileus and was discharged 102 days after admission. As of six months after discharge, she has had no recurrent IPO and has been able to continue her outpatient visits and attend a disability workshop. 

## Discussion

In this case report we describe a young female patient with MELAS who demonstrated rapid improvement of IPO with early initiation of TPN and no drug administration. Since then, the patient has received adequate nutritional management and there has been no IPO recurrence. In prior cases of IPO in patients with MELAS, parenteral nutrition was performed [4,12-14]. However, there are no reported cases of early introduction of TPN. The one case that improved with TPN did not demonstrate early improvement, as the patient had multiple episodes of IPO and an ileostomy was constructed [6]. There have been no reports suggesting that early TPN improved IPO.

In this case, we thought that TPN improved IPO because it was caused by a state of nutritional depletion due to anorexia, and nutritional therapy was successful because mitochondrial function in the intestinal tract remained relatively intact. Although it is not possible to demonstrate a causal relationship between the treatment of TPN and improvement of IPO based on this case alone, it might be highly likely that the pathophysiological mechanism of IPO in our patient was related to mitochondrial dysfunction and imbalance in energy metabolism [4]. If mitochondrial function remains intact in the intestinal tract, paralytic ileus might improve with nutritional therapy. 

TPN might improve IPO in patients with MELAS. Patients with MELAS with the m.3243A>G mutation have low ATP production in various organs due to mitochondrial dysfunction [15], so it is assumed that organ function is compromised when malnutrition occurs. One potential outcome is IPO [9]. In fact, many causes of death in patients with MELAS often stem from disorders in the heart and brain, organs that are characterized by high ATP consumption [16]. CIPO is rarely a direct cause of death [17]. However, there have been reports of deaths due to CIPO-induced aspiration pneumonia in parents and children with MELAS [5], and CIPO contributes to a poor prognosis in patients with MELAS. Low BMI has been shown to cause severe IPO in patients with MELAS [7]. In this case, the patient had a BMI of 12.6 kg/m2, which was sufficiently low to increase the risk of IPO.

In patients with MELAS, distigmine bromide [12] and neostigmine [13] have been shown to be effective for treating IPO, while prucalopride has demonstrated efficacy when the condition is characterized by acute onset [14]. There is one report of TPN being effective [6]. In both the present case and a previous report [6], the patients were followed conservatively with peripheral supplementation for IPO, but did not improve. In the previous report, peripheral venous replacement fluid and nutrients were administered through a nasogastric tube, but there was no improvement in bowel obstruction.

In that case, high-calorie infusion through a central venous catheter was started 19 days after onset at 1.5 times the basal metabolic rate, and on the following day the intestinal obstruction symptoms resolved. In the present case, TPN was started 10 days after onset using a low-calorie formula due to concerns about refeeding syndrome, but it took 10 days for the ileus to resolve. We thought that this might indicate the need for an adequate energy supply to improve IPO via TPN.

On the other hand, many patients with MELAS experiencing intestinal obstruction do not improve with TPN [5,9]. This discrepancy may be explained by differences in mitochondrial and smooth muscle function in the intestinal tract. In particular, myenteric plexus neuropathy and visceral myopathy with cyclooxygenase defects in smooth muscle are the most likely causes of IPO [4]. In patients with MELAS whose IPO improves with TPN, smooth muscle function may have been impaired due to nutritional deficiency, while the degree of neuropathy and visceral muscle damage may have been small. As a result, when the nutritional deficiency is treated by TPN, smooth muscle function recovers and IPO symptoms become less severe.

In this case, however, we did not evaluate the gastrointestinal tract, for instance by lower gastrointestinal endoscopy, or perform pathological examination, such as with intestinal tissue biopsy. Thus, a limitation of this case is that we did not determine the relationship between TPN and the status of both the myenteric plexus and the intestinal smooth muscle. In this case and the previous case mentioned above [6], IPO occurred against a background of malnutrition, but in other reported cases, IPO symptoms improved without TPN [14,18]. It remains unclear which patients with MELAS and IPO will derive benefit from TPN. 

Surgery for IPO has been reported to be effective in patients with normal small bowel motility [18]. In patients with IPO who have undergone surgery, the surgical procedure included exploratory laparotomy, bowel or colon resection (subtotal colectomy, left colectomy, ileocaecal resection, bowel resection, subtotal small bowel resection), venting gastrostomies, or terminal stomas [19]. In general, the prognosis after surgery for IPO is not good [7]. In this case, we considered surgical treatment when paralytic ileus developed, but opted for conservative follow-up because surgery in a young patient can lead to long-term complications such as adhesive ileus. In the previous case mentioned above, the patient developed IPO after undergoing ileal resection and subtotal colorectal resection, and while these symptoms improved with the initiation of TPN [6], the patient may have had good intestinal motility. A unique feature of the present case is that TPN improved IPO in a patient with MELAS who had never undergone intestinal surgery.

Insulin injections might be preferable for glycemic control in patients with MELAS who have had IPO. Glucagon-like peptide-1 (GLP-1) receptor agonists improve mitochondrial function in patients with MELAS whose insulin secretion is maintained [20]. Incretin-related drugs, particularly GLP-1 receptor agonists and DPP-4 inhibitors, were shown to decrease intestinal peristalsis more than sodium/glucose cotransporter 2 inhibitors [21], and are associated with an increased incidence of intestinal obstruction [22]. In particular, DPP-4 inhibitors were found to cause bowel obstruction more frequently than GLP-1 receptor agonists [11,22]. We consider that incretin-related medications should be avoided in patients with MELAS who have IPO. 

## Conclusions

Nutritional management with TPN might improve paralytic ileus in patients with MELAS and IPO. We believe that further case accumulation is needed to determine which patients truly benefit from early TPN initiation.
